# Liquid Biopsy Testing Can Improve Selection of Advanced Non-Small-Cell Lung Cancer Patients to Rechallenge with Gefitinib

**DOI:** 10.3390/cancers11101431

**Published:** 2019-09-25

**Authors:** Riziero Esposito Abate, Raffaella Pasquale, Alessandra Sacco, Maria Carmela Piccirillo, Alessandro Morabito, Paolo Bidoli, Giovanna Finocchiaro, Rita Chiari, Luisa Foltran, Roberta Buosi, Marcello Tiseo, Laura Giannetta, Ciro Battiloro, Gianpiero Fasola, Gianpiero Romano, Libero Ciuffreda, Antonio Frassoldati, Filippo de Marinis, Federico Cappuzzo, Nicola Normanno

**Affiliations:** 1 Cell Biology and Biotherapy Unit, Istiuto Nazionale Tumori “Fondazione G. Pascale”-IRCCS, 80131 Naples, Italy; r.espositoabate@istitutotumori.na.it (R.E.A.); raffaella.pasquale@hotmail.it (R.P.); a.sacco@istitutotumori.na.it (A.S.); 2 Clinical Trials Unit, Istituto Nazionale Tumori, IRCCS-Fondazione G. Pascale, 80131 Napoli, Italy; m.piccirillo@istitutotumori.na.it; 3 Medical Oncology, Thoracic Department, Istiuto Nazionale Tumori “Fondazione G. Pascale”-IRCCS, 80131 Napoli, Italy; a.morabito@istitutotumori.na.it; 4 Department of Oncology, Azienda Socio Sanitaria Territoriale (ASST) Monza, Presidio San Gerardo, 20900 Monza, Italy; p.bidoli@hsgerardo.org; 5 Department of Oncology & Hematology, Humanitas Clinical & Research Center, 20089 Rozzano (MI), Italy; giovanna.finocchiaro@cancercenter.humanitas.it; 6 Medical Oncology, Santa Maria della Misericordia Hospital, Azienda Ospedaliera di Perugia, University of Perugia, 06129 Perugia, Italy; rita.chiari@aulss6.veneto.it; 7 Department of Medical Oncology, Centro di Riferimento Oncologico di Aviano (CRO), IRCSS, 33081 Aviano, Italy; luisa.foltran@cro.it; 8 Department of Medical Oncology, Ospedale Santo Spirito, 15033 Casale Monferrato, Italy; rbuosi@aslal.it; 9 Department of Medicine and Surgery, University of Parma, 43121 Parma, Italy; mtiseo@ao.pr.it; 10 Niguarda Cancer Center, Grande Ospedale Metropolitano Niguarda, 20162 Milan, Italy; laura.giannetta@ospedaleniguarda.it; 11 Division of Pulmonary Oncology, Azienda Ospedaliera Dei Colli Monaldi, 80131 Naples, Italy; ciro.battiloro@ospedalideicolli.it; 12 Azienda Ospedaliera Universitaria Santa Maria della Misericordia, 33100 Udine, Italy; gianpiero.fasola@asuiud.sanita.fvg.it; 13 Presidio Ospedaliero Vito Fazzi, 73100 Lecce, Italy; oncologia.polecce@ausl.le.it; 14 AOU Città della salute e della Scienza di Torino, 10126 Turin, Italy; l.ciuffreda@libero.it; 15 Arcispedale S. Anna, Ferrara 44124, Italy; a.frassoldati@ospfe.it; 16 Division of Thoracic Oncology, European Institute of Oncology, IRCCS, 20122 Milan, Italy; filippo.demarinis@ieo.it; 17 Director Oncology and Hematology Department, AUSL Romagna, 48121 Ravenna, Italy; federico.cappuzzo@auslromagna.it

**Keywords:** non-small-cell lung cancer, rechallenge, resistance, liquid biopsy, p.T790M

## Abstract

The ICARUS trial is a phase II, open label, multicenter, single arm study conducted to investigate the efficacy, safety, and tolerability of a rechallenge treatment with the first-generation tyrosine kinase inhibitor (TKI) gefitinib in advanced non-small-cell lung cancer (NSCLC) patients carrying activating mutations of the epidermal growth factor receptor (*EGFR*). The ICARUS trial enrolled 61 patients who were rechallenged with gefitinib at progression after second-line chemotherapy. Serum-derived circulating cell-free DNA (cfDNA) collected before the rechallenge from a cohort of 29 patients, was retrospectively analyzed for the *EGFR* exon 19 deletions and for the p.L858R and p.T790M single nucleotide variants (SNV). The analysis of cfDNA detected the same *EGFR* activating mutation reported in the tumor tissue in 20/29 patients, with a sensitivity of 69%. Moreover, a p.T790M variant was found in 14/29 patients (48.3%). The median progression-free survival (PFS) was 2.7 months for p.T790M positive patients (CI 95% 1.4–3.1 months) versus 3.5 months for the p.T790M negative patients (CI 95% 1.6–5.3 months), resulting in a statistically significant difference (Long rank test *p* = 0.0180). These findings confirmed the role of the p.T790M mutation in the resistance to first-generation TKIs. More importantly, our data suggest that TKI rechallenge should be guided by biomarker testing.

## 1. Introduction

In the era of personalized medicine, molecular profiling has become essential in order to choose the most appropriate therapeutic strategy. This is particularly relevant for non-small-cell lung cancer (NSCLC) with adenocarcinoma histology. This subgroup of tumors carries a number of genetic alterations for which drugs approved for clinical use are available. Indeed, assessment of mutations in the epidermal growth factor receptor (*EGFR*) and *BRAF* genes, rearrangements of anaplastic lymphoma kinase *ALK* and of proto-oncogene tyrosine-protein kinase (*ROS1*), and expression of programmed death-ligand 1 (*PD-L1*) is mandatory for the choice of the appropriate therapies in patients with advanced NSCLC [1].

Approximately 10–15% of Caucasian NSCLC patients carry an activating *EGFR* mutation leading to a constitutive activation of the tyrosine kinase domain and downstream pathways involved in the tumor growth [2]. A number of clinical trials demonstrated that in advanced NSCLC patients carrying *EGFR* mutations, first-line treatment with EGFR tyrosine kinase inhibitors (TKIs) produces a higher response rate and a longer progression-free survival (PFS) as compared to standard chemotherapy [3,4]. Nevertheless, patients who initially respond to TKIs will relapse, usually within one year from the start of treatment [5].

Mechanisms of acquired resistance to EGFR TKIs are usually classified in three main groups [6]. Alterations of the EGFR leading to reduced activity of TKIs represent one of the major mechanisms of acquired resistance to these agents. In an additional fraction of tumors activation of alternative signaling pathways occurs, through genetic alterations or increased levels of expression of growth factors/growth factor receptors or intracellular signal transduction proteins. Finally, complex phenotypic transformations such as epithelial-to-mesenchymal transition or transformation into small-cell-lung cancer (SCLC) might also carry resistance to EGFR TKIs.

The most common mechanism of acquired resistance to first- and second-generation EGFR TKIs is the p.T790M point mutation of the *EGFR* gene that occurs in about 50% of tumors progressing after first- or second-generation TKIs [2,6]. This mutation is relatively rare in patients before TKI treatment, although the frequency of this phenomenon depends on the sensitivity of the techniques used for its detection. Recently, the third-generation EGFR TKI osimertinib became available for patients who progressed after a first line with a TKI and whose tumors carry a p.T790M mutation [7]. However, no alternative treatment exists for patients whose tumors developed other mechanisms of resistance.

Several clinical studies have assessed the possibility to rechallenge patients who progressed on first-generation TKIs after a course of chemotherapy. The hypothesis underlying these trials is that second-line chemotherapy might be effective on the clones of resistant cells thus restoring the sensitivity to EGFR TKIs. In this respect, the Iressa rechallenge in advanced NSCLC *EGFR*-M+ patients who responded to gefitinib used as first-line or previous treatment (ICARUS) trial investigated prospectively the safety and the efficacy of a rechallenge treatment with gefitinib in a cohort of advanced *EGFR*-mutant NSCLC patients [8]. Eligible patients had initially responded to first-line gefitinib and progressed after second-line chemotherapy and were treated with gefitinib as the treatment rechallenge. Of the 61 enrolled patients, 32 (52.5%) obtained a clinical benefit, with 3 (4.9%) achieving a partial response (PR) and 29 (47.5%) having stable disease (SD). Median progression-free survival (PFS) was 2.8 months, while median overall survival (OS) was 10.2 months. Overall, the results of this study indicated that gefitinib rechallenge offers modest benefit and represents an option only for patients for whom no other treatment is available.

In the ICARUS trial as well as in most trials of TKI rechallenge, patients received the rechallenge with gefitinib without any prospective evaluation of biomarkers. In this regard, we want to emphasize that this trial was run before the approval of osimertinib for patients progressing on first- or second-generation TKIs. At that time, information on T790M status of patients was not available, while this analysis is currently part of the routine diagnostic workout of patients recurring after treatment with TKIs.

The identification of patients who might benefit from the rechallenge with TKI remains a crucial issue. However, since in the large fraction of advanced or metastatic NSCLC patients a rebiopsy at progression of disease is not feasible, circulating cell-free DNA (cfDNA) analysis could represent a valid alternative for the molecular selection of these patients. In this study, we performed a retrospective *EGFR* molecular profiling of serum-derived cfDNA from patients enrolled in the ICARUS trial, thus evaluating the feasibility of cfDNA to identify patients suitable for the TKI rechallenge approach.

## 2. Results

### 2.1. Patients

The ICARUS trial enrolled 61 patients; of these, 58 received gefitinib. Serum samples were available for a subgroup of 29/58 (50%) advanced *EGFR*-mutant NSCLC patients treated within the ICARUS trial. The serum samples were collected at the enrollment in the study, i.e., before the retreatment with gefitinib in third-line. In particular, serum samples were available from 13 patients carrying the p.L858R point mutation in exon 21 and from 16 cases with an exon 19 deletion, as assessed by tissue testing at the initial diagnosis of lung cancer. The clinical and pathological characteristics of this subgroup are representative of the entire cohort previously described [8] (Table 1). 

### 2.2. Serum Analysis for EGFR Mutations

We first performed analysis of serum-derived cfDNA by using the Therascreen Plasma RGQ kit, which detected the same *EGFR* activating mutation found in tumor tissue at baseline in 13 out of 29 patients, showing therefore a 44.8% sensitivity. In particular, serum Therascreen analysis identified 6/13 (46.1%) p.L858R mutations and 7/16 (43.7%) exon 19 deletions, as shown in Table 2. Because of the poor sensitivity of the Therascreen test, we reassessed all the samples, including those already found positive with the Therascreen, by droplet digital PCR (ddPCR) that detected 20 *EGFR* mutations in the 29 serum samples, with a sensitivity of 69%. Furthermore, ddPCR confirmed all the mutations found by Therascreen on serum. In detail, ddPCR testing detected in 9/13 (69.2%) cases with a p.L858R mutations and in 11/16 (68.8%) patients with an exon 19 deletions the same *EGFR* variant found in tissue (Table 2). Analysis with ddPCR also allowed defining the allelic frequency of the *EGFR* mutations in the positive cases. The mean and the median allelic frequency of *EGFR* mutations (either exon 19 deletions or p.L858R) in samples positive at both Therascreen and ddPCR analyses were 7.13% and 5.73%, respectively (range 0.16–13.53%). In contrast, the mean and median value of *EGFR* mutation allelic frequency for samples positive only at ddPCR analysis were 0.35% and 0.17%, respectively (range 0.1–1%).

Notably, both Therascreen and ddPCR detected in the serum samples only the same *EGFR* variant previously identified in the tissue biopsy. In nine samples neither activating nor resistance mutation was detected by both techniques.

We observed similar findings for the p.T790M variant. Analysis of serum-derived cfDNA with the Therascreen kit revealed this variant in 2 out of 20 *EGFR* mutant serum samples. However, ddPCR analysis detected the presence of the resistance mutation p.T790M in 14 cases. We found no p.T790M mutations within the nine cases with no activating mutation in the serum. Overall, a p.T790M mutation was found in 14/29 (48.3%) patients at the enrollment in the ICARUS trial. The allelic frequency of the p.T790M variant in serum was generally much lower as compared with the exon 19 deletions and p.L858R, being <1% in all but one case.

### 2.3. PFS Analysis According to EGFR p.T790M Status in cfDNA

**Figure 1:**
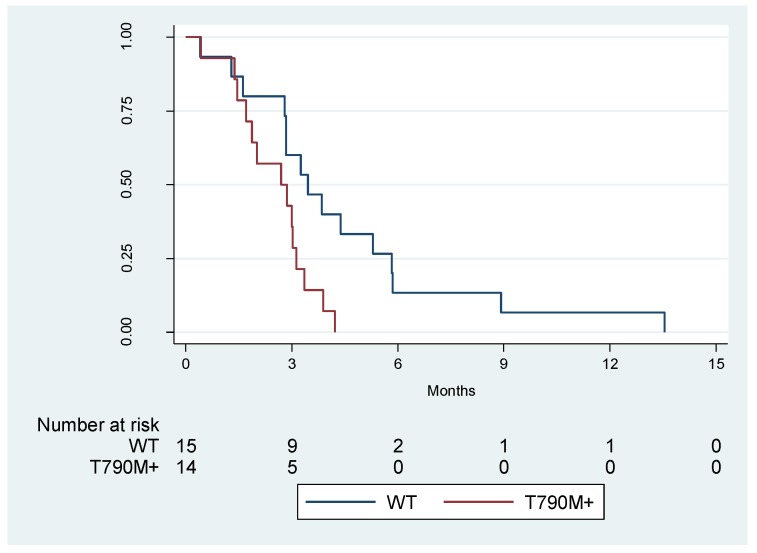
Kaplan-Meier survival estimates of progression free survival (PFS) of p.T790M negative (WT) and positive (T790M+) non-small-cell lung cancer (NSCLC) patients according to the droplet digital PCR (ddPCR) serum analysis.

In the cohort of 29 patients, the median PFS of patients with (*N* = 14) and without (*N* = 15) p.T790M mutation serum were retrospectively evaluated. Cases for which serum analysis was not able to identify either the sensitive mutation or the resistance mutation were included in the p.T790M wild-type group. The PFS was 2.7 months for p.T790M positive patients (CI 95% 1.4–3.1 months) versus 3.5 months for the p.T790M negative patients (CI 95% 1.6–5.3 months), resulting in a statistically significant shorter PFS in the group of patients with detectable levels of p.T790M mutation in cfDNA (Long rank test *p* = 0.0180) (Figure 1). At Cox regression analysis, patients with a p.T790M mutation had a hazard risk (HR) of progression of 2.72 when compared with p.T790M negative patients (CI 95% 1.15–6.45).

Median TKI-free interval (defined as the time elapsed from the end of first EGFR-TKI treatment to the start of the rechallenge therapy) was 8.6 months (interquartile (IQ) range 6.4–10.0) in p.T790M-negative patients and 5.5 months (IQ range 3.3–7.6) in p.T790M-positive cases (*p* = 0.004 calculated by Kruskal-Wallis rank test). In addition, median numbers of cycles of rechallenge with gefitinib was 5 (IQ range 3–7) in the overall population, and 7 (IQ range 5–11) and 3.5 (IQ range 2–5) in patients without or with the p.T790M mutation, respectively (*p* = 0.005 calculated by the Kruskal-Wallis rank test). We listed the detailed information on rechallenge with gefitinib for individual patients in Supplementary Table S1.

**Figure 2:**
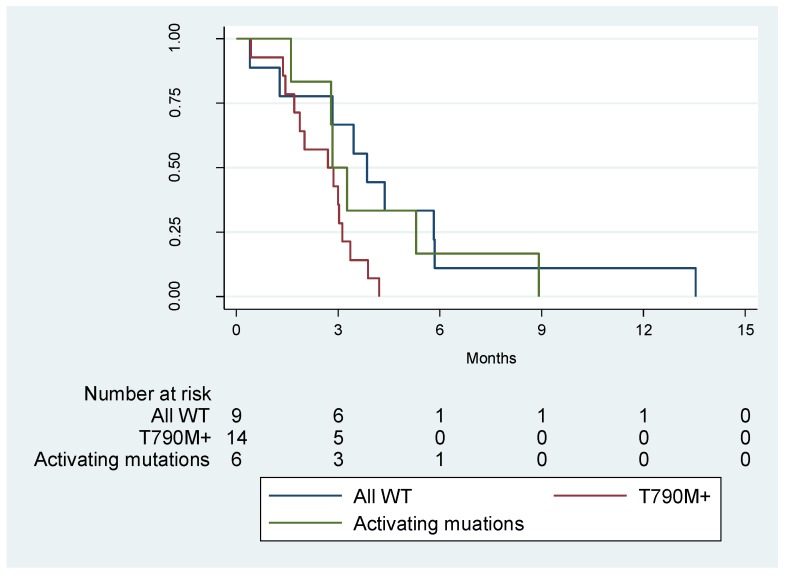
Kaplan-Meier survival estimates of progression free survival (PFS) of the three groups: Double positive (p.T790M and *EGFR* sensitizing mutation); single positive (only for the *EGFR* sensitizing mutation) and double negative (no *EGFR* mutations), according to digital droplet PCR (ddPCR) serum analysis.

Finally, dividing the cohort of 29 patients in three groups: serum-positive for *EGFR* sensitizing and p.T790M mutations (*N* = 14); serum-positive only for the *EGFR* sensitizing mutation (*N* = 6); serum-negative for all *EGFR* mutations (*N* = 9), we observed no statistically significant differences in PFS (*p* = 0.0546; Figure 2). 

However, the trend of the curves of both the double negative cases and negative cases only for the T790M is similar and differs from that of the double positive patients who in any case appear to have a worse prognosis.

## 3. Discussion

Rechallenge with TKI in NSCLC patients with *EGFR* mutations relies on the hypothesis that only a fraction of the *EGFR*-mutant tumor cells acquires a second genetic alteration that determines resistance. Actually, this supposition has been demonstrated in a number of studies showing that the p.T790M mutation is often heterogeneously expressed in tumors becoming resistant to first- or second-generation TKIs. The double mutant clone would have a growth advantage over the cells carrying only the original activating *EGFR* variant, determining the disease progression on TKI treatment. A subsequent line of chemotherapy would be highly effective on exponentially growing double-mutant clones. Therefore, their elimination would result in the subsequent expansion of cells with the only original *EGFR* mutation that, at this point, would be sensitive to TKIs. Sequist et al., have formally demonstrated this hypothesis at a molecular level in a seminal paper [9]. These authors showed that in selected patients the disappearance of the p.T790M resistant mutation following treatment with chemotherapy was indeed associated with the recovery of clinical sensitivity to EGFR TKIs.

Despite this strong biological hypothesis, the majority of prospective clinical trials showed a modest benefit from TKI rechallenge. Indeed, the response rate ranged between 0% and 22%, with median PFS ranging between 2 and 3.4 months [10,11,12]. These findings are in line with the results of the ICARUS trial that we previously described. Although some retrospective studies reported slightly better results, overall these findings suggest that rechallenge is an option only for patients for whom no other treatment option exists.

The major limit of the studies on rechallenge is that they did not evaluate prospectively any biomarker to select patients that might benefit from this therapeutic approach. In this respect, our study suggests that *EGFR* mutant NSCLC patients who progress after gefitinib and subsequent chemotherapy and do not have serum p.T790M do benefit more from the rechallenge with gefitinib as compared with p.T790M-positive patients. Because of the lack of alternative treatment strategies in patients who do not carry a p.T790M at progression of the disease, our data support the use of rechallenge with first-generation TKIs in p.T790M-negative patients.

Our findings have a number of biological and clinical implications. We could detect *EGFR* activating mutations in 69% of the cases. The sensitivity of *EGFR* mutation testing of liquid biopsy ranged between 40% and 80% in different studies, depending on the technology used for testing [13]. In particular, methods based on emulsion PCR, such as ddPCR and BEAMing, showed a higher sensitivity as compared with real time PCR-based approach, including the Therascreen assay. However, different variables might affect the sensitivity of the testing. First, it must be emphasized that the best results are usually obtained by testing cfDNA extracted from the plasma, while in our study we only had the serum available. Serum samples are sub-optimal for cfDNA testing because of the higher levels of non-tumor DNA [14]. In addition, the majority of studies comparing tissue and liquid biopsy have enrolled previously untreated patients. While we do not expect that following chemotherapy tumors might lose the expression of the *EGFR* mutation that is quite often a clonal event, the levels of cfDNA correlate with the tumor burden. In this respect, at least in some patients who are progressing after chemotherapy, we might expect a reduced tumor burden as compared with baseline.

In this study, we detected the p.T790M in almost 50% of the serum samples. It is difficult to compare these findings with previous reports in which the sensitivity for p.T790M testing in liquid biopsy was calculated by taking into account the p.T790M status of the tumor tissue. For example, Oxnard et al., found a sensitivity of plasma genotyping for detection of p.T790M with BEAMing of 70% among patients with a tissue biopsy positive for this variant. However, among patients with p.T790M-negative tumors, 31% of the cases were positive for the p.T790M in the liquid biopsy [15]. The frequency of the p.T790M mutation at progression after first- or second-generation TKIs ranges between 50% and 65% in different studies. Given the limits of sensitivity of liquid biopsy, our data suggest that chemotherapy did not eradicate the resistant clone in any of the patients who developed the p.T790M as mechanism of resistance. This finding does explain the limited activity of gefitinib rechallenge. The mean number of cycles of chemotherapy received by patients enrolled in the ICARUS trial was 4.7 ± 1.7 (range: 1–11), with 16 (26.2%) patients receiving ≤3 cycles and 45 (73.8%) >3 cycles. More importantly, the response rate was only 22.9%. We can hypothesize that in patients who did not respond to chemotherapy or who had a short exposure to cytotoxic drugs, no eradication of the p.T790M resistant clone might have occurred. In agreement with this hypothesis, patients with p.T790M-positive serum had a significantly shorter median TKI-free interval as compared with cases with p.T790M-negative serum. 

Our findings highlight the importance to assess the p.T790M status in patients who might be candidate to rechallenge with a first-generation EGFR TKI. In agreement with our results, Nakamura et al., reported that 4/5 patients with a history of p.T790M positivity in plasma-derived DNA had progression disease (PD) when rechallenged with a first-generation TKI. Interestingly, they also found that 3/4 patients who achieved PR with chemotherapy between treatments with EGFR TKIs, benefited from rechallenge. These findings further support the hypothesis that response to chemotherapy might be relevant to restore sensitivity to EGFR TKIs. In contrast, Zhang et al., found that the presence of the EGFR-resistance mutation p.T790M is not predictive of the clinical response to first-generation TKIs rechallenge [16]. Nevertheless, in this latter study, the analysis of the p.T790M was performed on tumor tissue by using Sanger sequencing in 70% of the cases and amplification refractory mutation system (ARMS) assay in 30%. Sanger sequencing has a very low sensitivity and it can easily result in false negative findings when assessing *EGFR* mutations [17]. This might be particularly relevant when testing for the EGFR p.T790M variant, which is often heterogeneous in TKI-resistant tumors [18]. In this respect, liquid biopsy testing has the advantage of recapitulating better the heterogeneity of the disease as compared with analysis of a single tumor biopsy specimen. Our results are also apparently different from the findings of Hata et al., who described a median PFS of 5.0 months for *EGFR*-mutant NSCLC patients with p.T790M and of 2.7 months for those without p.T790M in the tissue biopsy obtained before TKI rechallenge [19]. However, the definition of TKI rechallenge in the two studies was completely different. In the ICARUS study, patients received a chemotherapy treatment after the first-line gefitinib and before the rechallenge with the same drug within a prospective trial. In the retrospective study from Hata et al., patients who had received another TKI without TKI-free interval after initial TKI failure were categorized as patients who underwent TKI rechallenge [19]. Therefore, the two studies measured completely different phenomena.

An important point to discuss is that the p.T790M-negative population is actually a mixed group of patients. In fact, among these, there may be patients in whom chemotherapy has reversed the resistant phenotype. However, it could also contain patients who have a different resistance mechanism than p.T790M [20]. Unfortunately, we did not have sufficient cfDNA available to test with next-generation sequencing panels to identify the additional genetic alterations that might drive the resistance to EGFR TKIs. Finally, this cohort also contains the cases that did not have any *EGFR* mutation in the serum. Evidence suggests that this group of patients has an excellent prognosis and a more indolent course of the disease. In this regard, we would like to emphasize two aspects. The first is that literature data suggest that patients with the p.T790M mutation have a favorable course of the disease compared to those that do not develop this mutation [19,21]. In this respect, the observation that patients without the p.T790M did better in our study supports the hypothesis that gefitinib treatment could influence the course of the disease in this subgroup of patients while it was not effective in p.T790M-positive patients. The second is that the trend of the curves of double negative patients and negative patients only for p.T790M is similar and differs from that of patients with p.T790M. Therefore, we believe that the observed difference does not derive from a prognostic role of liquid biopsy but that the lack of p.T790M has a predictive role with respect to the possibility of rechallenge efficacy. Nevertheless, we do recognize the limits of our analysis that cannot formally distinguish between a prognostic and predictive value of the p.T790M because of the non-randomized nature of the study.

We do acknowledge that our study has several limitations. This is a retrospective analysis and serum samples were available only from 50% of the initial cohort of 58 patients treated within the ICARUS trial. In addition, the use of targeted sequencing panels might better define the presence of resistance mechanisms to EGFR TKIs.

## 4. Materials and Methods 

### 4.1. Study Design and Patient Population

The ICARUS trial (NCT01530334) is a phase II, open label, multicenter, single arm study conducted to investigate the efficacy, safety, and tolerability of a rechallenge treatment with oral gefitinib (250 mg/day) in 61 advanced *EGFR*-mutant NSCLC patients who responded to gefitinib in the first-line treatment and with a disease relapse after second-line chemotherapy. NSCLC tumor tissues were screened for *EGFR* mutations by standard diagnostic approaches in the participating centers. The trial was conducted in accordance with the provisions of the Declaration of Helsinki, Good Clinical Practice guidelines (as defined by the International Conference on Harmonisation), applicable regulatory requirements, and the policy on bioethics and human biologic samples of the trial sponsor, AstraZeneca. All patients provided written informed consent. This trial was funded by the sponsor and was designed by the principal investigators (Federico Cappuzzo, Filippo de Marinis, Nicola Normanno) and the sponsor. The sponsor was responsible for collection and analysis of data. The ethic committee of the IRCCS “Fondazione Pascale” approved this research on 11 January 2012 (n. 926).

### 4.2. Serum-Derived cfDNA 

Blood samples were collected into BD Vacutainer® 10-mL serum tubes with no additive (BD Diagnostics, Milan, Italy) at the recruitment time in the ICARUS trial before the rechallenge with TKI. The serum fraction was separated from the blood cells by centrifugation for 10 min at 4 °C at 3000 *g*. The collected serum was aliquoted and stored at −80 °C until use. cfDNA was extracted from 2 mL of serum using the QIAamp Circulating Nucleic Acid Kit (Qiagen, Crawley, UK) according to the manufacturers’ instructions. The cfDNA quantity was assessed with the dsDNA HS assay kit by the Qubit 2.0 Fluorometer (Thermofisher, Waltham, MA, USA).

### 4.3. Therascreen Plasma EGFR RGQ PCR Kit

Serum-derived cfDNA of all samples was analyzed for *EGFR* mutation status using a Scorpion ARMS-based EGFR mutation detection kit (Therascreen Plasma *EGFR* RGQ PCR kit; Qiagen, Crawley, UK), which detects exon 19 deletions and both the p. L858R and the p. T790M point mutations. Reactions were performed on the Rotor-Gene Q real-time PCR cycler (Qiagen, Crawley, UK). Run conditions and Ct values were calculated according to the manufacturers’ instructions.

### 4.4. Droplet Digital PCR Analysis 

Samples negative at Therascreen analysis were analyzed using the QX200 Droplet Digital PCR (ddPCR) System. We employed three ddPCR assays able to detect the most frequent alterations in the EGFR tyrosine kinase domain: the *EGFR* Exon 19 Deletions Screening Kit, designed to detect 15 deletions in exon 19 of the *EGFR* gene and other exon 19 deletions present in the region covered by primers; the EGFR p.L858R mutation assay; and the EGFR p.T790M mutation assay (Bio-Rad, Milan, Italy). The ddPCR reaction was performed as previously described [22] and data analysis was conducted using the QuantaSoft analytical software v1.7.4 (Bio-Rad, Milan, Italy). The cut-off sensitivity of the ddPCR test was set at 0.1%.

### 4.5. Statistical Analysis 

The Kaplan-Meier method was used to estimate the median progression free survival time (PFS), comparing the groups (cases without and with p.T790M point mutation) by log-rank test. Results were confirmed by Cox regression analysis. Statistical analyses were carried out using STATA MP/14.2.

## 5. Conclusions

Our findings do point out the need of molecular profiling to identify *EGFR*-mutant patients who might benefit of a rechallenge strategy. Our study also confirms that rechallenge is an option for those patients who do not carry a p.T790M variant and who have no alternative treatments available. In this respect, our data provide additional evidence that testing for p.T790M is mandatory to derive therapeutic decisions in patients progressing on first- and second-generation TKIs.

## Figures and Tables

**Table 1:** Clinical and pathological feature of the 29 patients included in the serum-derived circulating cell-free DNA (cfDNA) analysis as compared with the 61 patients enrolled in ICARUS trial.

Patient Characteristics	*N* = 61			*N* = 29	
Gender	Male	16	(26.2%)	9	(30.1%)
	Female	45	(73.8%)	20	(69.9%)
Age	Mean	67 years		68 years	
	Range	40–86 years		40–86 years	
WHO-PS	0	20	(32.8%)	11	(37.9%)
	1	30	(49.2%)	11	(37.9%)
	2	10	(16.4%)	6	(22.6%
	n.a.	1	(1.6%)	1	(1.6%)
Stage at study entry	IIIB	2	(3.3%)	2	(6.9%)
	IV	59	(96.7%)	27	(93.1%)
Smoking status	Never	41	(67.2%)	18	(62%)
	Current	3	(4.9%)	2	(6.9%)
	Former	16	(26.2%)	8	(27.6%)
	Missing	1	(1.6%)	1	(3.5%)
Chemotherapy response	CR	1	(1.6%)	0	(0%)
	PR	13	(21.3%)	10	(34.5%)
	SD	26	(42.6%)	10	(34.5%)
	PD	21	(34.3%)	9	(21%)

**Table 2:** Epidermal growth factor receptor (*EGFR*) mutations in serum-derived circulating cell-free DNA (cfDNA) samples obtained from non-small-cell lung cancer (NSCLC) patients enrolled in the ICARUS trial.

Tumor *EGFR* Mutational Status at Baseline, *n*		Serum-Derived cfDNA *EGFR* Mutational Status at Enrolment in the ICARUS Trial, *n*			
		Therascreen		droplet digital PCR (ddPCR)	
		Positive	Negative	Positive	Negative
**p.L858R**	13	6	7	9	4
**Del Exon 19**	16	7	9	11	5
**p.T790M**	-	2	27	14	15

## Supplementary Materials

The following are available online at https://www.mdpi.com/2072-6694/11/10/1431/s1, Table S1: Details on rechallenge with gefitinib for individual patients.

## References

[1] Planchard D., Popat S., Kerr K., Novello S., Smit E.F., Faivre-Finn C., Mok T.S., Reck M., Van Schil P.E., Hellmann M.D. (2018). Metastatic non-small cell lung cancer: ESMO Clinical Practice Guidelines for diagnosis, treatment and follow-up. Ann. Oncol..

[2] Schrank Z., Chhabra G., Lin L., Iderzorig T., Osude C., Khan N., Kuckovic A., Singh S., Miller R.J., Puri N. (2018). Current Molecular-Targeted Therapies in NSCLC and Their Mechanism of Resistance. Cancers.

[3] Davis C., Naci H., Gurpinar E., Poplavska E., Pinto A., Aggarwal A. (2017). Availability of evidence of benefits on overall survival and quality of life of cancer drugs approved by European Medicines Agency: Retrospective cohort study of drug approvals 2009–13. BMJ.

[4] Vyse S., Huang P.H. (2019). Targeting EGFR exon 20 insertion mutations in non-small cell lung cancer. Signal Transduct. Target Ther..

[5] Ke E.E., Wu Y.L. (2016). EGFR as a Pharmacological Target in EGFR-Mutant Non-Small-Cell Lung Cancer: Where Do We Stand Now?. Trends Pharmacol. Sci..

[6] Fenizia F., De Luca A., Pasquale R., Sacco A., Forgione L., Lambiase M., Iannaccone A., Chicchinelli N., Franco R., Rossi A. (2015). EGFR mutations in lung cancer: From tissue testing to liquid biopsy. Future Oncol..

[7] Mok T.S.K., Kim S.W., Wu Y.L., Nakagawa K., Yang J.J., Ahn M.J., Wang J., Yang J.C., Lu Y., Atagi S. (2017). Gefitinib Plus Chemotherapy Versus Chemotherapy in Epidermal Growth Factor Receptor Mutation-Positive Non-Small-Cell Lung Cancer Resistant to First-Line Gefitinib (IMPRESS): Overall Survival and Biomarker Analyses. J. Clin. Oncol..

[8] Cappuzzo F., Morabito A., Normanno N., Bidoli P., Del Conte A., Giannetta L., Montanino A., Mazzoni F., Buosi R., Burgio M.A. (2016). Efficacy and safety of rechallenge treatment with gefitinib in patients with advanced non-small cell lung cancer. Lung Cancer.

[9] Sequist L.V., Waltman B.A., Dias-Santagata D., Digumarthy S., Turke A.B., Fidias P., Bergethon K., Shaw A.T., Gettinger S., Cosper A.K. (2011). Genotypic and histological evolution of lung cancers acquiring resistance to EGFR inhibitors. Sci. Transl. Med..

[10] Kuiper J.L., Heideman D.A., Wurdinger T., Grunberg K., Groen H.J., Smit E.F. (2015). Rationale and study design of the IRENE-trial (NVALT-16): A phase II trial to evaluate iressa rechallenge in advanced NSCLC patients with an activating EGFR mutation who responded to an EGFR-TKI used as first-line or previous treatment. Clin. Lung Cancer.

[11] Asahina H., Oizumi S., Inoue A., Kinoshita I., Ishida T., Fujita Y., Sukoh N., Harada M., Maemondo M., Saijo Y. (2010). Phase II study of gefitinib readministration in patients with advanced non-small cell lung cancer and previous response to gefitinib. Oncology.

[12] Koizumi T., Agatsuma T., Ikegami K., Suzuki T., Kobayashi T., Kanda S., Yoshikawa S., Kubo K., Shiina T., Takasuna K. (2012). Prospective study of gefitinib readministration after chemotherapy in patients with advanced non-small-cell lung cancer who previously responded to gefitinib. Clin. Lung Cancer.

[13] Esposito Abate R., Pasquale R., Fenizia F., Rachiglio A.M., Roma C., Bergantino F., Forgione L., Lambiase M., Sacco A., Piccirillo M.C. (2019). The role of circulating free DNA in the management of NSCLC. Expert Rev. Anticancer Ther..

[14] Normanno N., Denis M.G., Thress K.S., Ratcliffe M., Reck M. (2017). Guide to detecting epidermal growth factor receptor (EGFR) mutations in ctDNA of patients with advanced non-small-cell lung cancer. Oncotarget.

[15] Oxnard G.R., Thress K.S., Alden R.S., Lawrance R., Paweletz C.P., Cantarini M., Yang J.C., Barrett J.C., Janne P.A. (2016). Association Between Plasma Genotyping and Outcomes of Treatment With Osimertinib (AZD9291) in Advanced Non-Small-Cell Lung Cancer. J. Clin. Oncol..

[16] Zhang Q., Ke E., Niu F., Deng W., Chen Z., Xu C., Zhang X., Zhao N., Su J., Yang J. (2017). The role of T790M mutation in EGFR-TKI re-challenge for patients with EGFR-mutant advanced lung adenocarcinoma. Oncotarget.

[17] Normanno N., Fenizia F., Castiglione F., Barberis M., Taddei G.L., Truini M., De Rosa G., Pinto C., Marchetti A. (2017). External quality assessment for EGFR mutations in Italy: Improvements in performances over the time. ESMO Open.

[18] Normanno N., Maiello M.R., Chicchinelli N., Iannaccone A., Esposito C., De Cecio R., D’Alessio A., De Luca A. (2017). Targeting the EGFR T790M mutation in non-small-cell lung cancer. Expert Opin. Ther. Targets.

[19] Hata A., Katakami N., Yoshioka H., Takeshita J., Tanaka K., Nanjo S., Fujita S., Kaji R., Imai Y., Monden K. (2013). Rebiopsy of non-small cell lung cancer patients with acquired resistance to epidermal growth factor receptor-tyrosine kinase inhibitor: Comparison between T790M mutation-positive and mutation-negative populations. Cancer.

[20] Santoni-Rugiu E., Melchior L.C., Urbanska E.M., Jakobsen J.N., Stricker K., Grauslund M., Sorensen J.B. (2019). Intrinsic resistance to EGFR-Tyrosine Kinase Inhibitors in EGFR-Mutant Non-Small Cell Lung Cancer: Differences and Similarities with Acquired Resistance. Cancers.

[21] Gaut D., Sim M.S., Yue Y., Wolf B.R., Abarca P.A., Carroll J.M., Goldman J.W., Garon E.B. (2018). Clinical Implications of the T790M Mutation in Disease Characteristics and Treatment Response in Patients With Epidermal Growth Factor Receptor (EGFR)-Mutated Non-Small-Cell Lung Cancer (NSCLC). Clin. Lung Cancer.

[22] Rachiglio A.M., Esposito Abate R., Sacco A., Pasquale R., Fenizia F., Lambiase M., Morabito A., Montanino A., Rocco G., Romano C. (2016). Limits and potential of targeted sequencing analysis of liquid biopsy in patients with lung and colon carcinoma. Oncotarget.

