# Supplementary material for: Liquid Biopsy Testing Can Improve Selection of Advanced Non-Small-Cell Lung Cancer Patients to Rechallenge with Gefitinib
Source: Cancers (Basel). 2019 Sep 25;11(10):1431. doi: 10.3390/cancers11101431 (PMC6826724; doi:10.3390/cancers11101431)
Supplement: Supplementary file 1 [file cancers-11-01431-s001.pdf]

Supplementary Materials: Liquid biopsy testing can improve selection of advanced non-small cell lung cancer patients to rechallenge with gefitinib

Supplementary table 1: Details on rechallenge with gefitinib for individual patients.

Patient ID	Rechallenge Treatment Start Date	Rechallenge Treatment End Date	Reason for interruption
03-01	27/12/2012	08/04/2013	Disease Progression
03-02	07/05/2013	31/01/2014	Disease Progression
04-01	04/06/2013	06/08/2013	Interrupted
04-02	29/07/2013	23/06/2014	Missing
04-03	23/08/2013	11/10/2013	Disease Progression
04-04	15/01/2014	26/02/2014	Disease Progression
05-01	19/10/2012	31/01/2013	Disease Progression
05-02	06/12/2012	12/12/2012	Patient died
05-03	06/06/2013	01/07/2014	Disease Progression
06-01	24/10/2012	14/01/2013	Interrupted
06-02	31/01/2013	24/04/2013	Clinical
09-02	30/01/2013	16/07/2014	Disease Progression
10-01	05/11/2012	14/06/2013	Disease Progression
10-02	16/01/2013	03/09/2013	Badly clinical condition
10-03	20/08/2013	10/01/2014	Disease
10-04	24/10/2013	04/11/2013	Patient ospitalized
11-01	23/07/2012	29/10/2012	Disease Progression
11-02	29/05/2013	11/11/2013	Disease Progression
11-03	12/12/2013	12/06/2014	Disease Progression
12-01	07/10/2013	12/05/2014	Disease Progression
15-02	26/07/2013	10/04/2014	Disease Progression
18-01	20/12/2012	24/04/2013	Disease Progression
18-02	10/06/2013	26/05/2014	Heart Attack
19-01	24/07/2013	23/08/2013	Missing
19-03	30/10/2013	20/11/2013	Disease Progression
21-01	20/12/2012	12/02/2013	Disease Progression
21-02	17/01/2013	18/04/2013	Disease Progression
21-04	30/05/2013	31/10/2013	Disease Progression
25-01	30/04/2013	16/08/2013	Disease Progression
